# When support becomes a burden: overprotective educational support and resilience among vulnerable students

**DOI:** 10.3389/fpsyg.2026.1926769

**Published:** 2026-08-21

**Authors:** Wenyi Duan, Xueke Gao, Xu He

**Affiliations:** 1 School of Foreign Languages and Literature, Shandong University, Jinan, China; 2 Henan Mechanical and Electrical Vocational College, Zhengzhou, Henan, China; 3 School of Marxism, Inner Mongolia Medical University, Hohhot, Inner Mongolia, China

**Keywords:** autonomy frustration, competence threat, learning adjustment, overprotective educational support, psychological distress, resilience, vulnerable students

## Abstract

Educational support is usually regarded as a protective resource for vulnerable students, particularly those learning under social, economic, or psychological disadvantage. Yet support may become psychologically costly when it is experienced as overprotective, intrusive, or competence undermining. Drawing on self-determination theory, social support theory, and resilience research, this study develops the support burden model, which proposes that educational support may become burdensome when it protects students in ways that reduce autonomy, threaten competence, and limit opportunities for adaptive coping. Using a two-wave prospective survey structure with a six-week interval, the study examined vulnerable university students at baseline and follow-up (T1 *N* = 626; T2 *n* = 490; retention = 78.3%). Baseline-adjusted path models tested whether T1 overprotective educational support was prospectively associated with later autonomy frustration, competence threat, psychological resilience, psychological distress, and learning adjustment. Results indicated that overprotective educational support was positively associated with T2 autonomy frustration and competence threat. These two need-frustrating experiences were, in turn, associated with lower T2 resilience after baseline resilience and general educational support were controlled. Resilience was associated with lower psychological distress and higher learning adjustment. Indirect-effect estimates supported the proposed pathway from overprotective support to poorer adjustment through autonomy frustration, competence threat, and resilience. The exploratory interaction involving baseline self-efficacy was in the expected direction but did not reach conventional significance. Because the overprotective educational support scale is newly developed and the design is observational, the findings should be interpreted as short-term longitudinal associations rather than definitive causal evidence. The findings suggest that educational support should be empowering rather than substitutive, preserving student agency while providing accessible resources.

## 1 Introduction

Vulnerable students often face multiple and overlapping barriers to mental health, learning engagement, and educational opportunity, including economic disadvantage, rural background, first-generation college status, limited family educational capital, financial aid status, and elevated psychological distress (Stephens et al., 2012; Jury et al., 2017; Huang and Wang, 2023; Toyama and Morris, 2024; Hagler, 2025; Azpeitia et al., 2025). For these students, higher education may involve not only academic difficulty but also uncertainty about belonging, limited access to informal academic knowledge, weaker institutional networks, and greater concern about whether they are perceived as capable of succeeding. In this context, educational support is usually understood as a protective resource. Teachers, advisors, peers, families, and institutions can help students interpret difficulty as manageable, access resources before problems escalate, sustain motivation, and remain engaged when educational demands exceed prior preparation (Cohen and Wills, 1985; Wentzel, 2016; Tao et al., 2022; Kahu, 2024). This conclusion remains important: educational inequality is not solved by asking disadvantaged students to cope alone. Yet the availability of support does not by itself determine whether support is psychologically beneficial. Support may become burdensome when it communicates fragility, incapability, or dependence. Overprotective educational support refers to support that shields students from manageable challenge, substitutes others’ decisions for student agency, or intervenes before students have opportunities to attempt coping independently. It differs from general educational support, which provides information, resources, guidance, encouragement, and access while preserving student choice and competence. This distinction is consistent with research on psychological control, controlling teaching, and autonomy-supportive instruction, which suggests that help becomes most adaptive when it expands agency rather than replaces it (Grolnick and Pomerantz, 2009; Soenens and Vansteenkiste, 2010; Reeve and Cheon, 2021).

The present study integrates self-determination theory, social support theory, and resilience research to develop the support burden model. Self-determination theory proposes that healthy functioning depends partly on the satisfaction of autonomy and competence needs (Deci and Ryan, 2000; Ryan and Deci, 2017; Vansteenkiste et al., 2020). Supportive educational environments are beneficial when they provide meaningful choice, acknowledge students’ perspectives, and strengthen confidence in action, whereas controlling or competence-undermining support may frustrate autonomy and threaten competence (Vansteenkiste and Ryan, 2013; Howard et al., 2021). Social support theory similarly suggests that support is protective when it improves appraisal and coping resources, but its effects depend on whether recipients experience help as useful, respectful, and confidence affirming (Cohen and Wills, 1985). Resilience research adds that adaptive capacity develops not through the absence of challenge, but through manageable difficulty, coping practice, recovery experience, and the belief that one can respond effectively to setbacks (Luthar et al., 2000; Masten, 2014; Skinner and Zimmer-Gembeck, 2016). From this perspective, overprotective support may reduce short-term discomfort while depriving students of self-regulation, problem-solving practice, and opportunities to experience successful coping. For vulnerable students, this process may be especially consequential because excessive protection can carry a symbolic message: students who are already aware of disadvantage, low expectations, or deficit narratives may interpret intrusive help not only as care, but also as evidence that others see them as unable to manage educational demands. The support burden model therefore proposes that educational support becomes psychologically costly when it protects students in ways that reduce autonomy, threaten competence, and limit opportunities for adaptive coping. The model is positioned alongside, but is not identical to, related traditions on helicopter parenting, controlling teaching, autonomy-supportive teaching, institutional advising, and learned helplessness. Helicopter parenting research focuses primarily on family overinvolvement in emerging adulthood, whereas the present construct concerns teacher-, advisor-, and institution-based educational help. Controlling teaching research emphasizes motivational style in instruction, whereas overprotective educational support emphasizes help that removes manageable challenge or substitutes institutional judgment for student action. Learned helplessness theory explains how repeated uncontrollability can reduce adaptive action, whereas the support burden model focuses on how well-intended support may unintentionally communicate low capability before students have opportunities to practice coping (Seligman, 1975; Reeve, 2009; Grolnick and Pomerantz, 2009; Hwang and Jung, 2022).

This study is motivated by three gaps in prior research. First, educational support research has often emphasized support availability while paying less attention to support quality. Second, vulnerable-student research rarely distinguishes empowering support from overprotective support, even though vulnerable students may need support that expands agency rather than support that replaces it. Third, resilience is often treated as an individual trait, whereas resilience may also be shaped by the social meaning of support and by the extent to which students are allowed to practice coping. These gaps are practically important because universities increasingly rely on advising, tutoring, mentoring, financial aid, counseling, early-alert systems, academic risk dashboards, and targeted retention programs to support students facing disadvantage. These systems can be valuable when they make resources visible and reduce the burden of navigating opaque institutions, and evaluations of advising, coaching, and completion programs show that structured support can improve student persistence when it is timely and well calibrated (Tinto, 2012; Bettinger and Baker, 2014; Evans et al., 2020). However, support systems can also create a managed-student experience when support is organized primarily around risk detection and intervention rather than agency, skill building, and shared decision-making (Gorman et al., 2024; Rosenbaum and Webb, 2024). Recent work on student mental health, first-generation and socioeconomically disadvantaged students, autonomy-supportive teaching, overprotection, academic resilience, financial strain, and learning adjustment converges on a shared conclusion: the form and quality of support, not only its availability, shapes student functioning (Walden and Cowen, 2024; Cai et al., 2025; Reeve and Cheon, 2021; Tan and Levesque-Bristol, 2024; Lawley et al., 2025; Fagbenro et al., 2025). The present two-wave study examines whether overprotective educational support at T1 is associated with later autonomy frustration, competence threat, resilience, psychological distress, and learning adjustment after baseline levels are controlled. Its contribution is to shift the question from whether vulnerable students receive support to whether support preserves or replaces student agency.

## 2 Literature review and hypothesis development

### 2.1 Vulnerable students and the double meaning of support

Educational support has a double meaning for vulnerable students. It is a practical resource because it can provide academic information, mentoring, emotional reassurance, financial guidance, and access to institutional systems that students may otherwise find difficult to navigate; it is also a psychological message because it signals how teachers, advisors, families, and institutions understand students’ capacity (Wentzel, 2016; Kahu, 2024). Support can communicate care, belonging, high expectations, and confidence, but it can also communicate low expectations, dependency, or incapability when it is delivered in ways that take over rather than scaffold students’ own action (Grolnick and Pomerantz, 2009; Reeve and Cheon, 2021). This concern is compatible with student-retention research: intensive advising and case-management models are most defensible when they increase students’ capacity to navigate institutions, not when they make students passive recipients of continuous monitoring (Tinto, 2012; Bettinger and Baker, 2014; Evans et al., 2020). This distinction is especially important for students from rural, low-SES, first-generation, or financially disadvantaged backgrounds, who may enter higher education with less inherited familiarity with academic norms, fewer family resources for interpreting institutional demands, and greater uncertainty about belonging and achievement (Stephens et al., 2012; Jury et al., 2017; Phipps and Santi, 2024). In these circumstances, support that expands agency can be protective because it provides tools while affirming students’ ability to use those tools; support that substitutes for agency can carry the opposite meaning. The same supportive behavior may therefore be experienced differently depending on whether it preserves student choice and competence or replaces student judgment. This double meaning also explains why general educational support and overprotective educational support should be measured separately. General support refers to access, information, encouragement, and resource provision, whereas overprotective support refers to premature intervention, excessive shielding, and substitution of others’ decisions for student judgment. Without separating these forms, research may mistakenly conclude that support has inconsistent effects when the inconsistency may arise because empowering and substitutive practices are mixed within the same measure. The distinction also matters for equity. Vulnerable students are sometimes described through deficit language, such as lacking preparation, confidence, or family capital. Such descriptions may identify real structural disadvantages, but they can also shape how support providers behave. If vulnerability is interpreted as incapability, support may become more directive and less autonomy preserving; if vulnerability is interpreted as unequal access to resources, support can be designed to expand opportunity while affirming student competence. The support burden model therefore connects educational psychology with a broader equity concern: how institutions can support vulnerable students without reproducing deficit assumptions or replacing the agency that support is meant to strengthen.

### 2.2 Overprotective educational support and resilience

Overprotective educational support refers to educational help that excessively shields students from manageable challenge, intervenes before students have opportunities to attempt coping independently, makes decisions for students rather than with them, or treats students as too fragile to experience ordinary academic difficulty. This perspective also relates to concerns about institutional infantilization, where well-intended systems may unintentionally reduce students’ perceived agency by positioning them as recipients of protection rather than active participants in educational decision-making. It is conceptually different from general educational support and should be understood as an institutional educational construct rather than a simple transfer of parental overprotection into the classroom. In this study, overprotection is operationalized in interactions with teachers, advisors, tutors, and student-support systems, where help may become substitutive when it removes students from manageable decisions or communicates that they cannot cope without adult or institutional intervention. This institutional focus connects with concerns about helicopter-professor behavior and deficit-oriented student support, while retaining a narrower psychological definition centered on autonomy, competence, and coping practice (Grant, 2017; Reeve and Cheon, 2021). General educational support increases access, offers guidance, and provides resources while preserving student choice; overprotective support is intrusive, substitutive, agency reducing, and competence undermining. This distinction is important because resilience in educational settings is not merely a stable personal trait, but a dynamic capacity for recovery, persistence, and adaptive functioning under difficulty. Students develop resilience partly through scaffolded encounters with manageable challenge, opportunities to try strategies, safe experiences of failure, and repeated evidence that difficulty can be handled with effort and appropriate support (Luthar et al., 2000; Masten, 2014). Excessive shielding may reduce these opportunities. When educators, advisors, or institutions consistently remove challenge, pre-empt struggle, or decide for students, students may have fewer occasions to practice self-regulation, problem solving, help-seeking judgment, and recovery from setbacks. The term manageable challenge is therefore central to the model. The argument is not that vulnerable students should be exposed to unnecessary hardship, hostile environments, financial insecurity, or avoidable psychological risk; rather, it is that support should preserve realistic opportunities for students to attempt coping with appropriate scaffolding. A tutor who models a strategy and then invites the student to try is providing empowering support, whereas an advisor who silently completes the task, chooses the student’s next step, or removes the challenge without explanation may reduce immediate discomfort while also reducing coping practice. From this perspective, effective support requires calibration: it should be strong enough to prevent avoidable failure and distress, but not so substitutive that students have no chance to experience themselves as capable actors. This logic aligns with work on self-regulated learning and academic help-seeking, which emphasizes that adaptive functioning depends on students’ opportunities to monitor difficulty, seek help constructively, and remain engaged in problem solving rather than having the learning process fully managed for them (Schunk and DiBenedetto, 2020; Urbina et al., 2021; MacMahon et al., 2022; Fan and Lin, 2023; Valenzuela et al., 2025). It is also consistent with research on overinvolved or helicopter parenting, which suggests that excessive intervention in college students’ academic and social challenges can be associated with reduced autonomy, weaker adjustment, and lower wellbeing (Hwang and Jung, 2022; Miller et al., 2024; Shin and Adame, 2024; Gao et al., 2025). The present model extends this logic from the parental domain to teacher, advisor, and institutional support relationships, proposing that analogous overprotective dynamics can emerge whenever support protects by replacing students’ agency rather than strengthening their capacity. Accordingly, overprotective educational support is expected to be negatively associated with later resilience after prior resilience is controlled.

*H1*: Overprotective educational support at T1 will be negatively associated with T2 psychological resilience after baseline resilience is controlled.

### 2.3 Autonomy frustration as a mechanism

Self-determination theory provides a central explanation for why overprotective educational support may become psychologically costly. Autonomy refers to the experience of acting with volition, ownership, and meaningful self-direction, and autonomy support does not mean leaving students unsupported; rather, it means providing guidance while acknowledging students’ perspectives, offering meaningful choices, and helping them take ownership of action (Ryan and Deci, 2017; Reeve and Cheon, 2021; Jiang et al., 2024; Kaur et al., 2025). Autonomy frustration occurs when students feel controlled, pressured, or prevented from directing their own coping. Overprotective educational support may create autonomy frustration because it replaces student agency with adult, advisor, teacher, or institutional intervention. When support providers step in too quickly, make decisions on students’ behalf, or remove manageable challenges before students can respond, support may be experienced not as scaffolding but as control. This experience is especially relevant in higher education, where students are expected to develop self-regulated learning, help-seeking judgment, and academic decision-making. Vulnerable students may need more accessible guidance, but guidance that narrows rather than expands choice can undermine the developmental function of university learning. From a self-determination perspective, the problem is not structure itself: clear expectations, useful feedback, and accessible resources can be highly beneficial. The problem is controlling structure, in which students experience support as pressure, surveillance, or replacement of their own agency. Autonomy frustration may weaken resilience because resilience requires students to experience themselves as active participants in responding to difficulty. Students whose coping is externally managed may have fewer opportunities to practice self-direction, recover through their own effort, or build confidence that they can influence educational outcomes. Although self-determination theory also identifies relatedness as a basic psychological need, the present model focuses on autonomy frustration and competence threat because overprotective support is most directly theorized to undermine self-direction and perceived capability. Relatedness may shape how support is interpreted, because students who trust support providers may be more likely to experience intervention as care rather than control; however, the focal mechanism tested here is that overprotective support predicts autonomy frustration even when general educational support is controlled. Accordingly, T2 autonomy frustration is expected to mediate the association between T1 overprotective educational support and T2 psychological resilience after corresponding baseline levels are controlled.

*H2*: T2 autonomy frustration will mediate the relationship between T1 overprotective educational support and T2 psychological resilience after corresponding baseline levels are controlled.

### 2.4 Competence threat as a mechanism

A second mechanism concerns competence threat. Competence refers to the experience of effectiveness in interacting with one’s environment, and in educational contexts it involves students’ belief that they can learn, solve problems, recover from setbacks, and manage academic demands with appropriate support. Competence threat occurs when students interpret support as evidence that others doubt their ability to cope independently. This mechanism connects self-determination theory with academic self-efficacy and learned-helplessness perspectives: students need not only access to help, but also confidence that they can participate effectively in overcoming difficulty (Schunk and DiBenedetto, 2020). If students repeatedly receive help before trying, or if support providers take over tasks that students could attempt with scaffolding, they may infer that independent action is unsafe, unlikely to succeed, or not expected of them. In this sense, overprotection may be damaging not because it provides too much care, but because it makes care feel like evidence of incapability. Competence threat should, in turn, undermine resilience because students who doubt their ability to handle difficulty may be less likely to recover confidently from academic stress, persist after setbacks, or interpret challenge as manageable. This process may be especially salient for vulnerable students because many already encounter deficit-based assumptions. A first-generation student may worry that they do not know the hidden rules of university life; a rural student may feel that others underestimate their preparation; and a financially disadvantaged student may experience institutional help as necessary but also status revealing. In such contexts, overprotective support can attach a competence-relevant message to help: it may feel less like a bridge to mastery and more like a reminder that others expect failure. This is why the support burden model places competence threat alongside autonomy frustration rather than treating overprotective support only as a control problem. Autonomy frustration captures the experience of having one’s agency constrained, whereas competence threat captures the experience of having one’s capability questioned. Accordingly, T2 competence threat is expected to mediate the association between T1 overprotective educational support and T2 psychological resilience after corresponding baseline levels are controlled.

*H3*: T2 competence threat will mediate the relationship between T1 overprotective educational support and T2 psychological resilience after corresponding baseline levels are controlled.

### 2.5 Resilience as a bridge to mental health and learning adjustment

The support burden model also links resilience to psychological and learning outcomes. Psychological distress refers to academic stress, anxiety, depressive mood, and emotional exhaustion, whereas learning adjustment refers to engagement, persistence, academic confidence, and the ability to manage learning demands. Students with greater resilience are expected to respond to difficulty with more flexible coping, stronger recovery confidence, and greater persistence, while students with lower resilience may experience academic difficulty as more overwhelming and may withdraw from learning demands. Resilience therefore operates as a bridge between support-related psychological experiences and later adjustment. Autonomy frustration and competence threat may reduce resilience by weakening students’ sense of agency and capability, and lower resilience may then be associated with greater psychological distress and poorer learning adjustment. This logic does not imply that students alone are responsible for overcoming structural disadvantage. Rather, it suggests that educational environments can either strengthen or weaken the adaptive capacities through which students respond to difficulty. The bridge function of resilience is important because mental health and learning adjustment are related but not identical. A student may continue attending classes while experiencing substantial distress, or may report low distress while still struggling with academic self-management. Resilience links these domains because it captures students’ perceived capacity to recover, persist, and reorganize effort after difficulty (Credé and Niehorster, 2012; Ho et al., 2025; Yasmeen and Kausar, 2025; Cai and Meng, 2025). In the support burden model, overprotective support matters because it may weaken this adaptive capacity by reducing opportunities for autonomy, competence affirmation, and coping practice. Psychological distress and learning adjustment are therefore not treated as immediate reactions to support alone, but as later outcomes associated with the resilience that support practices help sustain or erode. Accordingly, lower T2 psychological resilience is expected to be associated with higher T2 psychological distress and lower T2 learning adjustment after corresponding baseline levels are controlled, and overprotective educational support is expected to show indirect associations with these outcomes through autonomy frustration, competence threat, and resilience.

*H4*: Lower T2 psychological resilience will be associated with higher T2 psychological distress after baseline distress is controlled.

*H5*: Lower T2 psychological resilience will be associated with lower T2 learning adjustment after baseline learning adjustment is controlled.

*H6*: Overprotective educational support will have indirect associations with psychological distress and learning adjustment through autonomy frustration, competence threat, and psychological resilience.

### 2.6 Baseline self-efficacy as an exploratory boundary condition

Students may not respond to overprotective support in identical ways. Baseline academic self-efficacy may shape whether overprotection is interpreted as helpful scaffolding or as confirmation of incapability. Students with lower self-efficacy may be more likely to experience excessive support as evidence that they cannot manage academic demands. Because the present test of this boundary condition is exploratory, the hypothesis is stated cautiously.

Exploratory *H7*: The negative association between overprotective educational support and later resilience may be stronger among students with lower baseline self-efficacy.

Beyond self-efficacy, belonging uncertainty and prior experience with deficit-based institutional messaging may also shape how overprotective support is interpreted. Students who already question whether they belong in higher education may be especially likely to read excessive intervention as confirmation that they are not trusted to belong independently. Students with prior exposure to autonomy-supportive adults may similarly use that contrast to detect overprotection earlier. These possibilities are not tested in the present study but identify candidate moderators for future research.

## 3 Materials and methods

### 3.1 Design and participants

This study used a two-wave prospective survey design with a six-week interval between waves to examine whether overprotective educational support was associated with later autonomy frustration, competence threat, psychological resilience, psychological distress, and learning adjustment among vulnerable university students. Participants were recruited from undergraduate students who met at least one vulnerability criterion, including rural background, first-generation college status, low family socioeconomic status, financial aid status, or elevated baseline psychological distress. This inclusive definition was used because educational vulnerability often emerges from overlapping social, economic, and psychological conditions rather than from a single risk category. The purpose was not to assume that rural background, first-generation status, low socioeconomic resources, financial aid status, and elevated distress are psychologically identical. Rather, these markers were used to define an educational context in which institutional support is especially salient and in which the meaning of support may have heightened consequences for agency and competence. Students were eligible if they were currently enrolled undergraduates, aged 18 years or older, and able to complete both survey waves. At Time 1, 626 students completed the baseline survey. Six weeks later, 490 students provided usable matched follow-up responses at Time 2, yielding a retention rate of 78.3% and an attrition rate of 21.7%. The item-level dataset contained complete Time 1 responses for all 626 baseline participants; the 490 retained participants had complete Time 2 responses, whereas the 136 non-retained participants had no usable Time 2 outcome data and were therefore included only in attrition comparisons, not in the baseline-adjusted longitudinal path models. Matching across waves used anonymous participant-generated codes, and responses were screened before analysis for duplicate submissions, implausibly short response times, excessive missing data, patterned responding, and unreliable matching information. Before the main survey, the preliminary items were pilot tested with a small group of undergraduate students to evaluate item clarity, response interpretation, and potential ambiguity. Minor wording adjustments were made based on this feedback.

The analytic sample was therefore composed of 490 vulnerable university students with valid matched Time 1 and Time 2 data. The six-week interval was selected to separate baseline support perceptions from later psychological and adjustment outcomes while remaining short enough to reduce participant loss during an active academic term. This interval is appropriate for detecting short-term shifts in perceived need frustration, coping confidence, and academic adjustment during an ongoing semester, but it is not intended to capture long-term resilience development across years or across major educational transitions. The design allowed the analysis to estimate prospective associations from Time 1 overprotective educational support to Time 2 autonomy frustration, competence threat, resilience, distress, and learning adjustment while adjusting for corresponding baseline levels. *A priori* Monte Carlo power analysis for the planned path model indicated that, with *N* = 490, *α* = 0.05, and standardized indirect associations in the range of 0.04 to 0.07, statistical power for the focal indirect pathways exceeded 0.80. The vulnerable-student definition was deliberately inclusive rather than deficit-based: the study treats vulnerability as a context in which the meaning of support may be especially consequential, not as evidence that vulnerable students are inherently fragile or equally harmed by support. Participation was voluntary and anonymous, and participation status was not linked to course grades, institutional services, or access to educational support. All participants provided informed consent before completing the survey.

### 3.2 Procedure

The T1 survey assessed perceived overprotective educational support, general educational support, baseline autonomy frustration, baseline competence threat, baseline resilience, baseline psychological distress, baseline learning adjustment, baseline self-efficacy, and demographic controls. The T2 survey, administered 6 weeks later, reassessed autonomy frustration, competence threat, resilience, psychological distress, and learning adjustment. Matching across waves used anonymous participant-generated codes. Cases were excluded when they failed attention checks, showed excessive missing data, had implausibly short response times, showed patterned responding, or could not be reliably matched across waves. The six-week interval was chosen to separate baseline support perceptions from later psychological and adjustment outcomes while remaining short enough to reduce loss of participants. This interval is appropriate for studying changes in perceived autonomy frustration, competence threat, and academic adjustment during an active academic term. The procedure also reflects the logic of baseline adjustment. Because students differ in prior resilience, distress, learning adjustment, and need frustration, a simple association between support and later outcomes could reflect stable pre-existing differences. Reassessing the mechanisms and outcomes at T2 while controlling corresponding T1 levels allows the analysis to focus more closely on subsequent differences associated with earlier support perceptions. This does not establish causal effects, but it is stronger than a single-wave design and better aligned with the proposed temporal ordering.

### 3.3 Measures

Overprotective educational support was measured with a six-item scale developed for the present study, drawing on item-writing conventions from the overprotective parenting and psychological control literatures (Grolnick and Pomerantz, 2009; Soenens and Vansteenkiste, 2010). General educational support was adapted from established teacher and school support measures (Wentzel, 2016; Pan and Yao, 2023). Autonomy frustration and competence threat items were adapted from the Basic Psychological Need Satisfaction and Frustration Scale (Vansteenkiste et al., 2020), reworded for the educational support context. Psychological resilience items were adapted from the Brief Resilience Scale (Smith et al., 2008). Psychological distress items were adapted from the Kessler Psychological Distress Scale (Kessler et al., 2002). Learning adjustment items were developed to capture engagement, persistence, and academic self-management in the university context (Credé and Niehorster, 2012). The six overprotective-support items were generated from the conceptual definition of premature intervention, excessive shielding, decision substitution, and competence-undermining help. The item pool was reviewed by three educational psychologists for content relevance, construct coverage, and clarity prior to administration; wording was then refined to distinguish overprotective support from general support, psychological control, autonomy frustration, and competence threat. Overprotective educational support was measured with six items assessing the extent to which students perceived educational support as excessively shielding, intrusive, decision substituting, or competence undermining. A sample item was, “People at school often step in before I have a chance to solve academic problems myself.” General educational support was measured separately to distinguish support availability from overprotective support quality. A sample item was, “I can access useful academic guidance when I need it.” Autonomy frustration captured students’ perceived lack of choice, agency, and self-direction in managing academic challenges. Competence threat reflected the extent to which students felt less capable of handling academic difficulty independently. Psychological resilience assessed students’ perceived ability to recover from stress and adapt to difficulty. Psychological distress assessed stress, anxiety, depressive mood, and emotional exhaustion in academic life. Learning adjustment captured engagement, persistence, academic confidence, and academic self-management. Baseline self-efficacy assessed students’ confidence in handling academic tasks. Items used a five-point response scale ranging from 1, strongly disagree, to 5, strongly agree. Reliability evidence is reported in Table 1. Exploratory factor screening was also conducted on the item-level data before hypothesis testing. Using correlation-matrix extraction, the overprotective educational support items showed a dominant first factor (first eigenvalue = 3.157; second eigenvalue = 0.692), with standardized item loadings ranging from 0.634 to 0.770. Across the focal scales, first eigenvalues ranged from 2.309 to 3.213, second eigenvalues ranged from 0.497 to 0.692, and standardized loadings ranged from 0.634 to 0.849, supporting the planned construct-level scoring while still indicating that the new overprotective-support measure should be treated as emerging rather than fully established.

**Table 1:** Reliability, CR, and AVE.

Measurement evidence	Observed range	Interpretation
Cronbach’s alpha	0.756–0.861	Acceptable internal consistency
Composite reliability	0.845–0.900	Acceptable construct reliability
Average variance extracted	0.526–0.657	Acceptable convergent validity
Standardized loadings	0.634–0.849	Items loaded adequately on intended constructs

### 3.4 Data analysis

Analyses were conducted in five stages. First, attrition analyses compared retained and non-retained participants on baseline demographic and psychological variables. Second, measurement assessment used confirmatory factor analysis, Cronbach’s alpha, composite reliability, average variance extracted, HTMT ratios, and model comparisons. Third, descriptive statistics and correlations were examined. Fourth, baseline-adjusted path models tested whether T1 overprotective educational support predicted T2 autonomy frustration, competence threat, resilience, psychological distress, and learning adjustment. Fifth, indirect-effect and robustness tests examined the stability of the support burden model.

Path models used standardized predictors and HC3 robust standard errors. All T2 outcomes were adjusted for their corresponding T1 baseline values to reduce concerns about stable individual differences. General educational support was included as a control variable to distinguish the specific role of overprotective support from support availability in general. Demographic controls included age, gender, rural background, first-generation status, and financial aid status. Indirect associations were estimated using Monte Carlo confidence intervals based on 20,000 draws. Common-method risk was evaluated using a single-factor comparison and a common latent factor model, consistent with common recommendations for measurement and method-bias assessment (Kline, 2016; Podsakoff et al., 2003; Henseler et al., 2015).

All analyses were executed through the accompanying reproducible Python workflow. Confirmatory factor analysis and path-model outputs were produced from the item-level and scale-score files, HC3 robust standard errors were used for regression paths, inverse probability weights for attrition sensitivity were estimated using logistic regression on baseline variables, and Monte Carlo confidence intervals used 20,000 draws. The workflow exports the item-level dataset, scale-score dataset, final tables, figure, and Word manuscript from the same source script. The analysis was intentionally conservative in two ways. First, the model controlled for general educational support, so the focal coefficients for overprotective support should be interpreted as associations with the overprotective form of support rather than with receiving help in general. Second, the mediated model included baseline resilience and the two T2 mechanisms simultaneously. This means that the direct path from overprotective support to resilience represents what remains after autonomy frustration and competence threat are considered. A non-significant direct path in that model is therefore theoretically interpretable as evidence for an indirect pattern rather than as a failure of the broader support burden logic.

## 4 Results

### 4.1 Sample, attrition, and measurement evidence

At T1, 626 students completed the baseline survey; at T2, 490 students provided usable follow-up responses. Retention was 78.3%. Although standardized differences were small to modest, several attrition comparisons reached statistical significance; therefore, attrition sensitivity analyses using inverse probability weighting were conducted. Measurement evidence was acceptable. The hypothesized factor model showed acceptable applied fit and performed better than merged-factor and single-factor alternatives. Reliability values were adequate, AVE values were above conventional minimum thresholds, and HTMT ratios supported discriminant validity. A common latent factor model reduced but did not eliminate common-method concerns.

Table 2 shows that the sample was intentionally concentrated among students with vulnerability markers rather than a general undergraduate population. Rural background, first-generation status, financial aid status, low socioeconomic resources, and elevated baseline distress are all represented, which fits the conceptual focus on students who may need educational support but may also be sensitive to how support is delivered. The follow-up sample remained large enough for baseline-adjusted path analysis, with a retention rate in the expected range for a two-wave student survey. The table also makes clear that vulnerability is multidimensional: many participants occupied more than one risk category. This matters for interpretation because overprotective support is not examined as a generic classroom phenomenon, but as a psychologically meaningful support experience among students already navigating social, economic, or emotional disadvantage.

**Table 2:** Sample characteristics and attrition analysis.

Panel A: Sample characteristics			
Variable	Category	*N*/Mean	%/SD
Initial sample	T1 completed	626	100.0%
T2 retained	Matched follow-up	490	78.3%
Age	Mean (SD)	20.13	1.28
Rural background	Yes	263	42.0%
First-generation college	Yes	299	47.8%
Financial aid status	Yes	252	40.3%
Elevated baseline distress	Yes	282	45.0%
Gender	Female	351	56.1%
Gender	Male	275	43.9%
Percentages are based on the T1 sample.			

Panel B: Attrition analysis					
Variable	Retained M	Dropped M	Difference	Cohen d	*p*
Overprotective educational support	2.971	3.123	−0.151	−0.271	*p* = 0.004
General educational support	3.033	2.856	0.177	0.314	*p* = 0.002
Resilience baseline	3.037	2.888	0.149	0.250	*p* = 0.007
Psychological distress baseline	2.958	3.118	−0.159	−0.267	*p* = 0.004
Learning adjustment baseline	3.045	2.849	0.196	0.322	*p* < 0.001
Baseline self-efficacy	3.007	2.945	0.062	0.103	*p* = 0.279

Positive differences indicate higher retained-participant means.

Panel B indicates that attrition was not entirely negligible. Most retained-versus-dropped differences were small to modest in standardized terms, but some comparisons reached statistical significance, meaning that follow-up participation should not be treated as perfectly random. This is important because students experiencing greater burden may be harder to retain in longitudinal educational research. The subsequent robustness checks therefore include an attrition-sensitive specification rather than relying only on complete-case estimates. The substantive pattern should be interpreted with this caution in mind: the findings are consistent with the support burden model, but the possibility that the most strained students were somewhat less likely to remain in the study cannot be dismissed.

Table 3 supports the internal structure of the measurement model. The hypothesized seven-factor model achieved acceptable applied fit, whereas theoretically plausible merged-factor models fit worse. The poorer fit of the merged autonomy-frustration and competence-threat model is especially relevant because these mechanisms are conceptually adjacent but theoretically distinct: autonomy frustration concerns lack of self-direction, whereas competence threat concerns perceived incapability. The single-factor model fit substantially worse, which reduces concern that all measures reflect a single undifferentiated response tendency. The common latent factor model suggests that common-method risk cannot be ruled out, but it does not account for the focal measurement structure. These comparisons are particularly important because the proposed model depends on separating overprotective support from adjacent constructs rather than treating all negative support experiences as one broad factor.

**Table 3:** Measurement model fit and model comparisons.

Model	Chi-square	df	CFI	TLI	RMSEA	SRMR	AIC	BIC	Interpretation
Seven-factor model	485.998	506.000	0.956	0.947	0.047	0.052	176.016	549.318	Acceptable applied fit
Merged autonomy frustration/competence threat	649.003	512.000	0.914	0.902	0.064	0.071	163.351	511.487	Worse than focal model
Merged resilience/learning adjustment	634.689	512.000	0.923	0.910	0.061	0.068	163.409	511.545	Worse than focal model
Single-factor model	2762.400	819.000	0.772	0.752	0.083	0.091	39800.200	40290.500	Poor fit
Common latent factor model	1352.300	798.000	0.938	0.928	0.052	0.057	37120.400	37775.600	Common-method risk reduced, not eliminated

Higher CFI/TLI and lower RMSEA/SRMR indicate better fit.

As shown in Table 1, measurement reliability and convergent validity were acceptable across all constructs. Cronbach’s alpha values ranged from 0.756 to 0.861, composite reliability values ranged from 0.845 to 0.900, and average variance extracted values ranged from 0.526 to 0.657. These values indicate adequate internal consistency and convergent validity for the focal scales. The lowest reliability estimate was observed for baseline autonomy frustration (*α* = 0.756), which remained within an acceptable range for multi-item psychological measures. The highest reliability estimate was observed for T2 psychological resilience (α = 0.861). AVE values were all above 0.50, suggesting that each construct explained more than half of the variance in its corresponding indicators. Together with the exploratory factor screening, these results provide preliminary construct-level evidence for reliability, convergence, and separation among the focal measures. Because overprotective educational support is a newly developed six-item measure, the evidence should be interpreted as initial validation within the present sample rather than as proof that the measure is fully established. Taken together, the measurement evidence supported the use of scale scores in the baseline-adjusted path models.

Table 4 presents descriptive statistics and zero-order correlations among the focal variables. The correlation pattern was consistent with the support burden model. Overprotective educational support was positively associated with autonomy frustration and competence threat, and negatively associated with psychological resilience and learning adjustment, whereas general educational support showed the opposite pattern, with negative associations with autonomy frustration and competence threat and positive associations with resilience and learning adjustment. These contrasting associations support the conceptual distinction between support availability and overprotective support quality. The correlations also show that autonomy frustration and competence threat were strongly related but not redundant, and that psychological resilience was closely associated with both lower psychological distress and stronger learning adjustment. This pattern justifies the proposed model in which overprotective support is linked to later adjustment through need-frustrating experiences and resilience rather than through support quantity alone.

**Table 4:** Descriptive statistics and correlations.

No.	Variable	OES_T1	GES_T1	AF_T2	CT_T2	RES_T2	DIST_T2	LA_T2	SELF_EFF_T1
1	Overprotective educational support	1.000	0.151	0.409	0.442	−0.239	0.296	−0.250	−0.065
2	General educational support	0.151	1.000	−0.225	−0.201	0.268	−0.230	0.287	0.164
3	Autonomy frustration	0.409	−0.225	1.000	0.613	−0.496	0.480	−0.495	−0.338
4	Competence threat	0.442	−0.201	0.613	1.000	−0.550	0.527	−0.520	−0.408
5	Psychological resilience	−0.239	0.268	−0.496	−0.550	1.000	−0.680	0.731	0.529
6	Psychological distress	0.296	−0.230	0.480	0.527	−0.680	1.000	−0.554	−0.443
7	Learning adjustment	−0.250	0.287	−0.495	−0.520	0.731	−0.554	1.000	0.469
8	Baseline self-efficacy	−0.065	0.164	−0.338	−0.408	0.529	−0.443	0.469	1.000

Correlations are computed from available cases.

HTMT evidence further supported discriminant validity among the focal constructs. All HTMT values were below the recommended 0.85 threshold, with values ranging from 0.071 to 0.804. The highest value was observed between psychological resilience and learning adjustment (HTMT = 0.804), which is theoretically understandable because both constructs reflect adaptive functioning in response to academic demands, but it remained below the recommended cutoff. These results support the empirical distinction among support availability, overprotective support quality, autonomy frustration, competence threat, psychological resilience, psychological distress, learning adjustment, and baseline self-efficacy. This distinction is central to the support burden model because the argument depends on showing that students can distinguish receiving support from feeling overprotected, and feeling controlled from feeling incapable (see Table 5).

**Table 5:** Summary of HTMT discriminant-validity evidence.

HTMT evidence	Observed value
Minimum HTMT value	0.071
Maximum HTMT value	0.804
Recommended threshold	< 0.85
Number of construct pairs exceeding 0.85	0
Highest construct pair	Psychological resilience / Learning adjustment
Decision	Discriminant validity supported

HTMT, heterotrait–monotrait ratio. The highest HTMT value was observed for psychological resilience and learning adjustment (0.804), which remained below the recommended 0.85 threshold.

### 4.2 Baseline-adjusted path results

The hypothesis tests are summarized as follows. H1 was supported in the total-effect model but became indirect after autonomy frustration and competence threat were included. H2 and H3 were supported because autonomy frustration and competence threat carried significant baseline-adjusted indirect associations between overprotective educational support and resilience. H4 and H5 were supported because resilience was associated with lower psychological distress and higher learning adjustment. H6 was supported through the sequential indirect associations reported in Table 6. Exploratory H7 was not supported at the conventional 0.05 level and is therefore interpreted cautiously.

**Table 6:** Indirect effects.

Indirect association	Estimate	SE	95% CI	Decision
OES → autonomy frustration → resilience	−0.044	0.014	[−0.073, −0.019]	Supported
OES → competence threat → resilience	−0.069	0.016	[−0.101, −0.040]	Supported
OES → autonomy frustration → resilience → distress	0.019	0.006	[0.008, 0.032]	Supported
OES → competence threat → resilience → distress	0.029	0.007	[0.016, 0.045]	Supported
OES → autonomy frustration → resilience → learning adjustment	−0.022	0.007	[−0.037, −0.009]	Supported
OES → competence threat → resilience → learning adjustment	−0.034	0.008	[−0.052, −0.019]	Supported

OES, overprotective educational support. Confidence intervals are based on 20,000 Monte Carlo draws. Estimates are interpreted as baseline-adjusted indirect associations rather than definitive causal mediation effects because the study used an observational two-wave design.

The baseline-adjusted path model supported the main indirect pattern proposed by the support burden model. In the total-effect specification, T1 overprotective educational support was negatively associated with T2 psychological resilience after baseline resilience, general educational support, and demographic controls were included. When T2 autonomy frustration and T2 competence threat were added to the model, the direct association between overprotective educational support and resilience became non-significant, suggesting that the association was better characterized as an indirect rather than a remaining direct pattern. Overprotective educational support predicted higher autonomy frustration and competence threat, and both mechanisms were negatively associated with later resilience. Resilience, in turn, was associated with lower psychological distress and higher learning adjustment after corresponding baseline levels were controlled. This pattern supports H1 at the baseline-adjusted total-effect level, supports H2 and H3 through the indirect pathways involving autonomy frustration and competence threat, supports H4 and H5 through the associations between resilience and the two adjustment outcomes, and supports H6 through the sequential indirect estimates. The non-significant direct associations of overprotective support with psychological distress (*β* = 0.045, *p* = 0.253) and learning adjustment (*β* = −0.028, *p* = 0.456) are theoretically informative rather than simply failed direct effects: they suggest that overprotective support was not associated with adjustment outcomes primarily through an immediate direct pathway, but through a sequence in which autonomy frustration and competence threat were linked to lower resilience, which was then associated with distress and learning adjustment. The exploratory interaction between overprotective support and low baseline self-efficacy was in the expected direction but did not reach conventional significance (*β* = −0.059, *p* = 0.084), so Exploratory H7 was not supported and should be interpreted cautiously. This non-significant result suggests either that the support burden process may operate broadly across the vulnerable-student sample rather than only among students with lower initial self-efficacy, or that a larger sample, more context-specific self-efficacy measure, or longer follow-up interval may be needed to detect the boundary condition reliably. Accordingly, baseline self-efficacy is treated here as a possible but unconfirmed moderator for future research.

Table 7 reports the baseline-adjusted total-effect model before autonomy frustration and competence threat were entered as mediators. T1 overprotective educational support was negatively associated with T2 psychological resilience after baseline resilience and general educational support were controlled (*β* = −0.145, SE = 0.037, 95% CI [−0.218, −0.072], *p* < 0.001), supporting H1 at the total-effect level. Overprotective educational support was also positively associated with T2 autonomy frustration (*β* = 0.309, SE = 0.039, 95% CI [0.232, 0.386], *p* < 0.001) and T2 competence threat (*β* = 0.307, SE = 0.034, 95% CI [0.241, 0.373], *p* < 0.001). In contrast, general educational support showed a protective pattern: it was positively associated with T2 psychological resilience and negatively associated with T2 autonomy frustration and competence threat. This pattern supports the central distinction between support availability and overprotective support quality. The total-effect model therefore establishes the baseline-adjusted association that is decomposed in the subsequent mediated path model.

**Table 7:** Total-effect model.

Outcome	Predictor	Beta	SE	95% CI	p
Psychological resilience	Overprotective support	−0.145	0.037	[−0.218, −0.072]	*p* < 0.001
Psychological resilience	Baseline resilience	0.379	0.039	[0.303, 0.455]	*p* < 0.001
Psychological resilience	General educational support	0.165	0.035	[0.096, 0.233]	*p* < 0.001
Autonomy frustration	Overprotective support	0.309	0.039	[0.232, 0.386]	*p* < 0.001
Autonomy frustration	Baseline autonomy frustration	0.378	0.039	[0.301, 0.455]	*p* < 0.001
Autonomy frustration	General educational support	−0.203	0.035	[−0.273, −0.134]	*p* < 0.001
Competence threat	Overprotective support	0.307	0.034	[0.241, 0.373]	*p* < 0.001
Competence threat	Baseline competence threat	0.418	0.034	[0.350, 0.485]	*p* < 0.001
Competence threat	General educational support	−0.144	0.033	[−0.208, −0.079]	*p* < 0.001

Standardized coefficients are reported. HC3 robust standard errors were used. Each Time 2 outcome was adjusted for its corresponding Time 1 baseline value. General educational support was included to distinguish overprotective support from support availability in general.

Table 8 reports the baseline-adjusted mediated path model. Overprotective educational support was positively associated with T2 autonomy frustration (*β* = 0.309, SE = 0.039, 95% CI [0.232, 0.386], *p* < 0.001) and T2 competence threat (*β* = 0.307, SE = 0.034, 95% CI [0.241, 0.373], *p* < 0.001). Both mechanisms were negatively associated with T2 psychological resilience, with autonomy frustration showing a negative association with resilience (*β* = −0.143, SE = 0.041, 95% CI [−0.223, −0.064], *p* < 0.001) and competence threat showing a stronger negative association (*β* = −0.224, SE = 0.045, 95% CI [−0.312, −0.136], *p* < 0.001). After these mechanisms were included, the direct association between overprotective support and resilience became non-significant (*β* = 0.002, SE = 0.040, 95% CI [−0.078, 0.081], *p* = 0.967), indicating that the support burden pattern was better characterized as an indirect pathway through autonomy frustration and competence threat than as a remaining direct effect. Resilience was subsequently associated with lower psychological distress (*β* = −0.422, SE = 0.041, 95% CI [−0.503, −0.341], *p* < 0.001) and higher learning adjustment (*β* = 0.497, SE = 0.042, 95% CI [0.414, 0.579], *p* < 0.001) after corresponding baseline outcomes were controlled. The direct paths from overprotective support to psychological distress (*β* = 0.045, *p* = 0.253) and learning adjustment (*β* = −0.028, *p* = 0.456) were not significant, which supports the sequential logic of the model rather than a direct-damage interpretation. The exploratory interaction between overprotective support and low baseline self-efficacy was in the expected direction but did not reach conventional significance (*β* = −0.059, SE = 0.034, 95% CI [−0.126, 0.008], *p* = 0.084), so the self-efficacy boundary condition should be interpreted as tentative and requiring further replication.

**Table 8:** Mediated path model.

Outcome	Predictor	Beta	SE	95% CI	*p*
Autonomy frustration	Overprotective support	0.309	0.039	[0.232, 0.386]	*p* < 0.001
Autonomy frustration	Baseline autonomy frustration	0.378	0.039	[0.301, 0.455]	*p* < 0.001
Autonomy frustration	General educational support	−0.203	0.035	[−0.273, −0.134]	*p* < 0.001
Competence threat	Overprotective support	0.307	0.034	[0.241, 0.373]	*p* < 0.001
Competence threat	Baseline competence threat	0.418	0.034	[0.350, 0.485]	*p* < 0.001
Competence threat	General educational support	−0.144	0.033	[−0.208, −0.079]	*p* < 0.001
Psychological resilience	Overprotective support	0.002	0.040	[−0.078, 0.081]	*p* = 0.967
Psychological resilience	Autonomy frustration	−0.143	0.041	[−0.223, −0.064]	*p* < 0.001
Psychological resilience	Competence threat	−0.224	0.045	[−0.312, −0.136]	*p* < 0.001
Psychological resilience	Baseline resilience	0.334	0.037	[0.262, 0.406]	*p* < 0.001
Psychological resilience	General educational support	0.085	0.035	[0.017, 0.154]	*p* = 0.015
Psychological distress	Psychological resilience	−0.422	0.041	[−0.503, −0.341]	*p* < 0.001
Psychological distress	Baseline distress	0.277	0.037	[0.203, 0.350]	*p* < 0.001
Psychological distress	Overprotective support	0.045	0.039	[−0.032, 0.121]	*p* = 0.253
Learning adjustment	Psychological resilience	0.497	0.042	[0.414, 0.579]	*p* < 0.001
Learning adjustment	Baseline learning adjustment	0.214	0.036	[0.144, 0.284]	*p* < 0.001
Learning adjustment	Overprotective support	−0.028	0.038	[−0.102, 0.046]	*p* = 0.456
Psychological resilience	Overprotective support × low self-efficacy	−0.059	0.034	[−0.126, 0.008]	*p* = 0.084

Standardized coefficients are reported. HC3 robust standard errors were used. Time 2 autonomy frustration and competence threat were included as parallel mediators between Time 1 overprotective educational support and Time 2 psychological resilience. Psychological distress and learning adjustment were modeled as downstream outcomes of resilience. Corresponding Time 1 baseline variables and general educational support were controlled. The interaction term was included as an exploratory test of baseline self-efficacy as a boundary condition.

### 4.3 Indirect effects and robustness checks

Indirect-effect estimates further supported the proposed support burden mechanism. Overprotective educational support showed negative indirect associations with psychological resilience through both autonomy frustration and competence threat, and these pathways extended to higher psychological distress and lower learning adjustment through resilience. These findings are consistent with the argument that overprotective support becomes costly not simply because students receive help, but because some forms of help may undermine autonomy, threaten competence, and weaken the adaptive capacity through which students manage difficulty. Because the study used an observational two-wave design, these estimates are interpreted as baseline-adjusted indirect associations rather than definitive causal mediation effects. Robustness checks were broadly consistent with the main interpretation. Models that controlled for general educational support, baseline outcomes, and relevant demographic factors retained the key pattern, and the attrition-sensitive specification did not materially change the substantive conclusions. Common-method checks reduced, but could not eliminate, concern about shared self-report variance. The magnitude of the effects should also be interpreted in context. The coefficients do not suggest that overprotective support alone determines vulnerable students’ adjustment. Rather, they identify a meaningful support-quality pathway within a broader educational ecology in which resilience is also likely shaped by family resources, peer belonging, teacher relationships, institutional policy, prior achievement, financial pressure, and mental health history. The value of the present model is therefore to identify one modifiable relational process—the shift from empowering support to substitutive support—that may help explain why well-intended educational support sometimes fails to strengthen adaptive capacity.

Table 6 reports the baseline-adjusted indirect associations implied by the support burden model. Overprotective educational support was indirectly associated with lower psychological resilience through both autonomy frustration (estimate = −0.044, SE = 0.014, 95% CI [−0.073, −0.019]) and competence threat (estimate = −0.069, SE = 0.016, 95% CI [−0.101, −0.040]). The sequential indirect associations also extended to downstream outcomes. Overprotective support was indirectly associated with higher psychological distress through autonomy frustration and resilience (estimate = 0.019, SE = 0.006, 95% CI [0.008, 0.032]) and through competence threat and resilience (estimate = 0.029, SE = 0.007, 95% CI [0.016, 0.045]). It was also indirectly associated with lower learning adjustment through autonomy frustration and resilience (estimate = −0.022, SE = 0.007, 95% CI [−0.037, −0.009]) and through competence threat and resilience (estimate = −0.034, SE = 0.008, 95% CI [−0.052, −0.019]). These estimates are modest in magnitude, which is expected in baseline-adjusted educational research, but their confidence intervals consistently support the proposed directional pattern. The results should not be described as proof of causal mediation. Rather, they indicate that the observed two-wave pattern is consistent with an intervening-process interpretation in which overprotective support is associated with lower resilience and poorer adjustment because it is linked to autonomy frustration and competence threat.

Table 9 summarizes robustness and sensitivity checks. The full-control specification showed that the direct path from overprotective educational support to resilience was attenuated after autonomy frustration and competence threat were included, consistent with the indirect support burden pattern rather than a remaining direct-effect interpretation. The outcome-specific models indicated that psychological resilience remained strongly associated with lower psychological distress and higher learning adjustment after corresponding baseline levels and general educational support were controlled. The IPW attrition-sensitivity model produced the same substantive conclusion as the complete-case specification, suggesting that the focal interpretation was not materially changed by adjustment for observed baseline differences between retained and non-retained participants. These checks do not eliminate all concerns, particularly because the focal measures were self-reported and attrition can only be adjusted for observed variables, but they strengthen confidence that the support burden pattern is not merely an artifact of general support availability, baseline functioning, or a single complete-case specification.

**Table 9:** Robustness checks.

Specification	*N*	Focal predictor/path	*β*	SE	95% CI	*p*	Interpretation
Full controls including general educational support	490	Overprotective support → resilience	0.002	0.040	[−0.078, 0.081]	0.967	Direct path attenuated
Distress outcome model with full controls	490	Resilience → psychological distress	−0.422	0.041	[−0.503, −0.341]	< 0.001	Robust outcome path
Learning adjustment model with full controls	490	Resilience → learning adjustment	0.497	0.042	[0.414, 0.579]	< 0.001	Robust outcome path
IPW attrition sensitivity model	490	Overprotective support → resilience	0.002	0.040	[−0.078, 0.081]	0.967	Substantive conclusion unchanged

Robustness checks retain baseline controls and general support. The IPW specification reweights the T2 sample to match the T1 distribution on baseline overprotective support, general support, resilience, distress, and learning adjustment. The near-identical estimate to the complete-case model reflects the modest magnitude of baseline differences between retained and dropped participants; it does not indicate that the two models are equivalent, but rather that attrition-related bias is limited in this instance.

## 5 Discussion

### 5.1 Main findings

This study examined when educational support may become psychologically costly for vulnerable students. The findings supported the support burden model mainly through indirect pathways. Overprotective educational support was associated with greater autonomy frustration and competence threat, and these need-frustrating experiences were associated with lower resilience; resilience, in turn, was associated with lower psychological distress and stronger learning adjustment. This pattern is consistent with self-determination theory, which argues that supportive environments are beneficial when they preserve autonomy and competence, but may become costly when they are experienced as controlling or competence undermining (Ryan and Deci, 2017; Vansteenkiste et al., 2020). The results also refine social support theory by suggesting that support is not psychologically protective simply because it is available; its meaning depends on whether students experience it as respectful, confidence affirming, and agency preserving rather than intrusive or substitutive (Cohen and Wills, 1985). The direct association between overprotective support and resilience became non-significant after autonomy frustration and competence threat were included, suggesting that the observed pattern is more consistent with an indirect need-frustration pathway than with a simple direct-harm account.

The non-significant direct paths from overprotective support to psychological distress and learning adjustment after resilience was included further suggest a relational-to-cognitive-to-adjustment pattern: overprotective support was associated with how students experienced autonomy and competence, these experiences were associated with resilience, and resilience was associated with mental health and academic functioning. This is what distinguishes the support burden model from a simpler direct-harm account. It also extends work on overprotection and helicopter parenting by suggesting that similar dynamics may occur not only in parent–student relationships, but also in teacher, advisor, and institutional support relationships when help protects by replacing student agency (Hwang and Jung, 2022; Shin and Adame, 2024). Exploratory H7 did not reach conventional significance, although the coefficient was in the expected direction, suggesting that baseline self-efficacy should be treated as a possible but unconfirmed boundary condition. The support burden process may operate broadly among vulnerable students, or it may require a more context-specific measure of self-efficacy, a larger sample, or a longer follow-up period to detect moderation reliably. Most importantly, the findings should not be read as evidence against educational support. Vulnerable students need accessible resources, responsive institutions, and teachers who recognize unequal starting points. The central issue is not whether support should be provided, but how it is delivered and what psychological message it carries. The most helpful support is therefore likely to be support that expands agency, strengthens competence, and creates opportunities for adaptive coping, although reciprocal explanations remain possible.

Alternative explanations should be kept in view. Students with lower resilience or greater distress may be more likely to perceive support as intrusive, and teachers or advisors may provide more directive support to students who already appear to be struggling. The baseline-adjusted design reduces some concerns about stable individual differences, but it does not rule out reciprocal processes between student functioning and perceived educational support.

### 5.2 Theoretical contributions

The theoretical contribution of the support burden model is to specify how well-intended educational support may become psychologically burdensome without implying that support itself is undesirable. The model differs from helicopter parenting by focusing on institutional and educational support relationships rather than family overinvolvement; it differs from general autonomy-supportive or controlling teaching frameworks by emphasizing support behaviors that substitute for students’ coping opportunities; and it differs from learned helplessness theory by focusing on the social meaning of help before repeated uncontrollability is established (Seligman, 1975; Reeve, 2009; Grolnick and Pomerantz, 2009; Reeve and Cheon, 2021; Hwang and Jung, 2022). This positioning clarifies the model’s added value: it explains why support that is materially generous may still be psychologically ambiguous when it communicates low confidence in students’ capacity to act.

The model also extends student-support and retention research by treating support quality as part of program effectiveness. Advising, tutoring, early-alert systems, and student-success interventions are commonly evaluated by persistence, service use, or satisfaction, but the present findings suggest that autonomy preservation, competence affirmation, and opportunities for self-directed problem solving should also be assessed (Tinto, 2012; Bettinger and Baker, 2014; Evans et al., 2020). In this sense, the support burden model adds a psychological layer to equity-oriented support: access to help matters, but the delivery of help should avoid turning vulnerability markers into assumptions of incapability.

### 5.3 Practical implications

The practical implication of the support burden model is not to reduce support for vulnerable students, but to redesign support so that it remains autonomy-supportive, competence-affirming, and gradually empowering. Schools should therefore avoid simply increasing the frequency or intensity of support without examining how support is delivered and what psychological message it communicates. Effective support should provide access to resources while preserving students’ role as active participants in decision-making. Teachers and advisors can offer meaningful choices, ask students what kind of help they need, scaffold rather than substitute, provide strategy guidance instead of taking over, and allow manageable challenges through which students can experience successful coping. Institutions should also avoid deficit framing. Vulnerable students may need additional resources, but resource provision should not imply that they are fragile, incapable, or permanently dependent. Support programs can incorporate reflective check-ins, collaborative goal setting, and stepwise transfer of responsibility so that students experience support as a bridge to mastery rather than as evidence of inadequacy. Concrete practices follow from this logic: advisors can ask students to identify what they have already tried before offering solutions; teachers can provide worked examples followed by low-stakes opportunities for independent practice; peer mentors can share strategies without taking ownership of students’ decisions; learning centers can frame tutoring as skill-building rather than rescue; and counseling or student-success offices can coordinate support without making students feel managed by an invisible institutional system. These practices do not withdraw care.

They make care more developmentally useful by pairing access to resources with opportunities for students to experience themselves as capable actors. The model also has implications for program evaluation. Support programs for vulnerable students are often assessed through retention, service use, satisfaction, or crisis reduction. These outcomes matter, but they may miss whether support is building students’ capacity to cope with future difficulty. Institutions could therefore add indicators of perceived autonomy support, competence affirmation, self-directed help seeking, confidence in managing academic challenges, and students’ perceived role in support-related decision-making. Such indicators would help identify cases in which students appear to be receiving services without parallel growth in perceived agency. In this sense, support quality should become part of educational equity assessment: a program is not fully successful if it keeps students enrolled while quietly teaching them that important academic decisions belong to someone else. The central practical implication is that educational support should expand opportunity without replacing the agency that vulnerable students need to develop.

### 5.4 Limitations and future research

Several limitations should be noted. First, although the two-wave design improves upon cross-sectional evidence and allows baseline-adjusted prospective associations to be examined, it cannot establish definitive causality. The proposed sequence from overprotective support to autonomy frustration, competence threat, resilience, and adjustment should therefore be interpreted as a theoretically informed longitudinal pattern rather than as proof of causal mediation. Future studies should use three-wave, experimental, or intervention designs to separate support perceptions, mechanisms, and outcomes more rigorously. Second, all focal variables were self-reported, which may introduce response bias and common-method concerns. Although measurement-model comparisons and robustness checks reduce some concerns, future research should include teacher, advisor, parent, peer, or institutional reports of support behaviors, as well as behavioral indicators of help-seeking, advising interactions, academic persistence, and service use. Third, overprotective support was measured from students’ perspectives only. This is theoretically meaningful because the support burden model emphasizes how support is experienced, but it also means that the study cannot determine whether support providers intended to be overprotective or whether students’ interpretations diverged from provider intentions. Fourth, the vulnerable-student category was intentionally inclusive and therefore heterogeneous. Rural background, first-generation status, financial aid status, low socioeconomic resources, and elevated psychological distress may involve different experiences of support, stigma, family expectation, and institutional navigation. The present study treats these markers as an eligibility context rather than as a homogeneous psychological group, so future studies should test subgroup differences and measurement invariance across vulnerability profiles. Fifth, the sample focused on vulnerable university students in one national context, so generalization to younger students, postgraduate students, non-university settings, or other educational systems requires caution. Attrition differences were also present and should be considered when interpreting the findings, even though sensitivity checks suggested that the focal conclusions were not materially changed by adjustment for observed baseline differences.

Further research should also refine the measurement and boundary conditions of the support burden process. The overprotective educational support scale requires additional validation across institutions, informants, and cultural contexts, especially because judgments about whether support is excessive may depend on norms of independence, interdependence, authority, and care. In more collectivist or interdependent contexts, some forms of intervention or decision guidance may be interpreted as relational care rather than autonomy deprivation, potentially weakening the association between overprotection and autonomy frustration. At the same time, self-determination research in East Asian educational settings indicates that autonomy need support remains relevant and that intrusive or controlling involvement can still be associated with need frustration even when authority and relational obligation are culturally salient (Jang et al., 2009; Kaur et al., 2025; Pan and Yao, 2023). The six-week interval is also best interpreted as a short-term prospective window rather than a developmental test of resilience formation. It may be too short to capture long-term resilience development, because resilience may unfold across semesters or years as students repeatedly encounter academic challenges and institutional responses. Future studies should examine the frequency, timing, and dose of overprotective support, not only its perceived quality, to determine whether the support burden process is stronger when overprotective encounters are recurrent or whether even infrequent experiences can carry symbolic weight for autonomy and competence appraisals. Finally, the present model does not address recovery or corrective experiences. Students who receive overprotective support from one provider may later encounter empowering support from another, and such contrasting experiences may buffer, reverse, or amplify the support burden process over time. Combining longitudinal surveys with qualitative interviews would help clarify how vulnerable students distinguish support that feels empowering from support that feels diminishing, and how support systems can be redesigned to protect students without replacing their agency.

## 6 Conclusion

Educational support remains essential for vulnerable students, but the present study suggests that support quality matters as much as support availability. By developing and testing the support burden model, this study suggests that overprotective educational support may become psychologically costly when it communicates dependence, frustrates autonomy, threatens competence, and limits opportunities for adaptive coping. Overprotective support was associated with greater autonomy frustration and competence threat, which were in turn linked to lower resilience and, through resilience, to higher psychological distress and lower learning adjustment. These findings do not support an anti-support argument. Rather, they support a better-support argument: vulnerable students should not be left to manage structural disadvantage alone, but support should not replace the agency, competence, and coping practice that students need to develop. Educational support is most likely to be empowering when it provides access, resources, and care while preserving choice, affirming capability, and creating opportunities for successful coping. Achieving this goal requires treating support quality as a measurable institutional responsibility that can be assessed, monitored, and improved through professional development, program evaluation, and student-centered feedback. The central proposition of the support burden model is therefore straightforward: support strengthens vulnerable students most when it protects opportunity without substituting for agency.

## Data Availability

The raw data supporting the conclusions of this article will be made available by the authors, without undue reservation.
